# 3D printed porous β-Ca_2_SiO_4_ scaffolds derived from preceramic resin and their physicochemical and biological properties

**DOI:** 10.1080/14686996.2018.1471653

**Published:** 2018-07-16

**Authors:** Shengyang Fu, Wei Liu, Shiwei Liu, Shichang Zhao, Yufang Zhu

**Affiliations:** a School of Materials Science and Engineering, University of Shanghai for Science and Technology, Shanghai, China; b Department of Orthopedics, Shanghai Sixth People’s Hospital, Shanghai Jiaotong University, Shanghai, China; c Shanghai Innovation Institute for Materials, Shanghai, P. R. China; d Hubei Key Laboratory of Processing and Application of Catalytic Materials, College of Chemical Engineering, Huanggang Normal University, Huanggang City, Hubei Province, China

**Keywords:** β-Ca_2_SiO_4_, preceramic resin, scaffolds, 3D printing, bone tissue engineering, 30 Bio-inspired and biomedical materials, 211 Scaffold / Tissue engineering / Drug delivery, 107 Glass and ceramic materials

## Abstract

Silicate bioceramic scaffolds are of great interest in bone tissue engineering, but the fabrication of silicate bioceramic scaffolds with complex geometries is still challenging. In this study, three-dimensional (3D) porous β-Ca_2_SiO_4_ scaffolds have been successfully fabricated from preceramic resin loaded with CaCO_3_ active filler by 3D printing. The fabricated β-Ca_2_SiO_4_ scaffolds had uniform interconnected macropores (ca. 400 μm), high porosity (>78%), enhanced mechanical strength (ca. 5.2 MPa), and excellent apatite mineralization ability. Importantly, the results showed that the increase of sintering temperature significantly enhanced the compressive strength and the scaffolds sintered at higher sintering temperature stimulated the adhesion, proliferation, alkaline phosphatase activity, and osteogenic-related gene expression of rat bone mesenchymal stem cells. Therefore, the 3D printed β-Ca_2_SiO_4_ scaffolds derived from preceramic resin and CaCO_3_ active fillers would be promising candidates for bone tissue engineering.

## GRAPHICAL ABSTRACT

**Figure:**
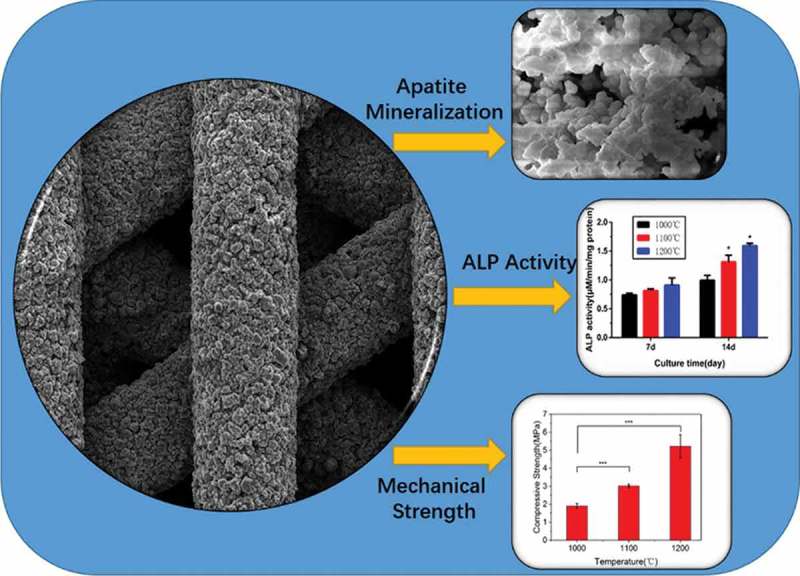

## 1. Introduction

Polymer-derived ceramics (PDCs), which are fabricated from preceramic polymers after sintering in a non-oxidative atmosphere (nitrogen or argon), have become attractive in the last two decades. One of the most important advantages is the possibility of combining the shaping and synthesis of ceramics. Preceramic polymers could be shaped by conventional plastic-forming techniques, such as casting, injection molding, warm pressing, and fiber drawing [1]. However, the PDCs normally contain a number of cracks or pores due to the large gas generation and shrinkage during the polymer-to-ceramic conversion [2,3]. Previous studies demonstrated that it is possible to control the shrinkage and obtain relatively dense and crack-free components by simply adding active fillers to preceramic polymers [2]. The active fillers play an important role in maintaining size or structure and participating in a reaction with preceramic polymers or atmosphere. Silicone resins are widely used as preceramic polymers and the resins can provide an amorphous silica-rich solid residue with the form of SiOC over 800 °C [4]. Thus, a common strategy is that oxide fillers react with the decomposition products of preceramic polymers to produce the desired ceramics. For example, Bernardo et al. prepared mullite (3Al_2_O_3_·2SiO_2_) and forsterite (2MgO∙SiO_2_) by using a silicone resin loaded with γ-Al_2_O_3_ and MgO nanoparticles, respectively [5–7]. In addition, ternary systems such as akermanite (2CaO·MgO·2SiO_2_) [8], hardystonite (2CaO·ZnO·2SiO_2_) [9], gehlenite (2CaO∙Al_2_O_3_∙SiO_2_) [10], and cordierite (2MgO∙2Al_2_O_3_∙5SiO_2_) [11] were also derived from silicone resin loaded with two active fillers.

Recently, silicate-based bioceramics have received significant attention in bone tissue engineering due to the ability for regulating the cell behaviors [12–17]. β-Ca_2_SiO_4_ ceramic is one of the bioactive ceramics [18] and can significantly induce the formation of hydroxyapatite layer on its surface in simulated body fluid (SBF) [19,20], showing the excellent bioactivity. Dai et al. [18] prepared porous β-Ca_2_SiO_4_ by sol–gel method, which showed good mineralization ability and cytocompatibility. Gou et al. [21–23] reported that β-Ca_2_SiO_4_ had a moderate degradation, and the dissolved ions could enhance the proliferative response of fibroblasts.

Generally, 3D porous scaffolds provide more advantages to repair bone defects, because an ideal 3D scaffold with a highly interconnected macroporous structure facilitates cell migration, nutrient delivery, bone ingrowth, and eventually vascularization [24–29]. To date, β-Ca_2_SiO_4_ scaffolds were prepared by conventional methods such as polyurethane foam templating [18] and porogen templating [30], which were not easy to control porous scaffolds with well pore interconnection, proper pore size, or high porosity [31,32]. 3D printing technique can control the architectures of porous scaffolds via computer-assisted design or computer-aided manufacturing. In the past several years, 3D printing technique has been widely used in fabricating biomaterials for bone tissue engineering combined with polymer-derived technique [33–41]. In this way, Bernardo et al. [37–39] prepared 3D wollastonite-based scaffolds from preceramic polymers. Calcium carbonate and commercial available silicone were selected as active filler and silicon source, respectively. Green scaffolds were shaped by 3D printing and wollastonite formed after sintering at 900 °C. Moreover, the addition of bioactive glass could control the solubility in Tris-HCl buffer, and the mechanical strength was around 6 MPa with a high porosity of 80%. Similarly, Fiocco et al. [40] fabricated silica-bonded calcite scaffolds by direct ink writing, starting from a paste comprising a silicone resin and calcite powders. The fabricated silica-bonded calcite scaffolds had open porosity of 56–64% and compressive strength of 2.9–5.5 MPa, depending on the geometry. Elsayed et al. [41] used silicone resin, CaCO_3_, and Mg(OH)_2_ as raw materials to prepare wollastonite-diopside glass-ceramic scaffolds by direct ink writing, which featured regular geometries and a high compressive strength (3.9–4.9 MPa) at a porosity of 68–76%. However, there are no reports on the fabrication of β-Ca_2_SiO_4_ scaffolds from preceramic polymers by 3D printing for bone tissue engineering.

In this study, we propose a facial strategy for the fabrication of porous β-Ca_2_SiO_4_ ceramic scaffolds from preceramic polymers loaded with CaCO_3_ active filler by 3D printing. A printable paste was prepared by dissolving silicone resin in isopropyl alcohol and followed the mixing of CaCO_3_ active filler. After 3D printing, green scaffolds were sintered at 900–1200 °C to obtain 3D porous β-Ca_2_SiO_4_ scaffolds. The effects of sintering temperature on the physicochemical and biological properties of the β-Ca_2_SiO_4_ scaffolds were systemically investigated.

## 2. Materials and methods

### 2.1 Materials

A commercial silicone resin (polysilsesquioxane, Silres®MK) was purchased from WackerChemie (Munich, Germany). Calcium carbonate (CaCO_3_, ≥ 99.0%) and isopropyl alcohol (IPA, ≥99.7%) were purchased from Sinopharm Chemical Reagent Co. Ltd (Shanghai, China).

### 2.2 3D printing of CaCO_3_/silicone scaffolds and ceramization

In this study, the 4th 3D Bioplotter™ (EnvisionTEC GmbH, Gladbeck, Germany) was used to print CaCO_3_/silicone scaffolds. Before printing, the injectable paste was prepared as follows. First, the CaCO_3_ powders were sieved through 400 mesh sieves to obtain powders with the particle size less than 38 µm. Subsequently, the silicone resin was completely dissolved in isopropyl alcohol and the sieved CaCO_3_ powders were added to obtain a Ca/Si molar ratio of 2:1. The mixture was stirred at room temperature to form the injectable paste. Finally, the prepared injectable paste was introduced into a plastic injection cartridge that was fixed onto the 3D printing device.

Simultaneously, rectangle models (8 × 8 × 8 mm^3^) were loaded into the Bioplotter CAD/CAM software and the scaffolds were plotted layer by layer, through the extrusion of the paste as a fiber, up to 25 layers. The architecture was changed by plotting fibers with 0/60/120 angle steps between successive layers; the dosing pressure to the syringe pump was 2.0–5.0 bar, the speed of the dispensing unit was 3.0–4.2 mm/s, and the nozzle size was 0.4 mm. The printed scaffolds were stored in a drying oven at 37 °C. Finally, all green scaffolds were sintered at different temperatures (900 ^o^C, 1000 ^o^C, 1100 ^o^C, and 1200 ^o^C) for 3, 5, and 7 h with a 2 °C/min heating rate in argon atmosphere.

### 2.3 Characterization

Wide-angle X-ray diffraction (XRD) patterns were recorded with a Bruker D8 ADVANCE X-ray powder diffractometer (Bruker Corp., Billerica, MA, USA). Scanning electron microscopy (SEM) was performed with a FEI Quanta 450 field emission scanning electron microscope. The compressive strength of the scaffolds was tested using a static materials testing machine (2.5 kN) (Zwick Roell, Ulm, Germany) at a crosshead speed of 0.5 mm/min. The porosity and density of the scaffolds was measured using Archimedes’ principle and water was used as liquid medium. The porosity (*P*) was calculated according to the following formulation: *P* = (*W*_sat_ − *W*_dry_)/(*W*_sat_ − *W*_sus_) × 100%, and the density was counted according to the formula: *ρ* = *W*_dry_ × (*ρ*_water_ − *ρ*_air_)/(*W*_dry_ − *W*_sat_) + *ρ*_air_, where *W*_dry_ is the dry weight of the scaffolds, *W*_sus_ is the weight of the scaffolds suspended in water, *W*_sat_ is the weight of the scaffolds saturated with water, *ρ*_water_ is the density of water (1.0 g/cm^3^), and *ρ*_air_ is the density of air (0.0012 g/cm^3^).

### 2.4 Degradation and the apatite mineralization ability of scaffolds

All the ceramic scaffolds were incubated into a freshly made SBF at a ratio of 1 g scaffold per 200 ml SBF at 37 ^o^C over a period of 28 days. The pH value of the SBF solution and the weight of dried scaffolds were recorded with changing SBF per 7 days. SEM and energy dispersive spectrometry (EDS) were used to observe the surface morphology and composition of all testing scaffolds after soaking in SBF for 7 days.

### 2.5 Cell adhesion and proliferation of rat bone mesenchymal stem cells (rBMSCs) on scaffolds

rBMSCs were first seeded onto the scaffolds at a density of 2 × 10^5^ cells per scaffold. After cultured for 3 days, the scaffolds were rinsed by phosphate buffer saline (PBS) three times. Subsequently, 2.5–3.0% glutaraldehyde was added for 15 min for fixation, and alcohol at concentration of 50%, 75%, 80%, 90%, 95%, and 100% were used for dehydration for 10 min twice. Finally, the samples were treated with isoamyl acetate overnight. The scaffolds were sprayed with gold for the observation with scanning electron microscope. On the other hand, the cell distributions on the surface of scaffold were also observed by fluorescence microscope. After cultured for 3 days, the culture medium was removed and the live cells were fixed in 4% paraformaldehyde before washing three times with PBS. Next, cells were treated with 0.5% Triton X-100 for 15 min. Afterward, the cell actin and nuclei were labeled with 0.6 ml of 5 mg/ml phalloidin (Yeasen, Shanghai) and 0.6 ml of 10 mg/ml 4′,6-diamidino-2-phenylindole (DAPI; Yeasen, Shanghai) for 5 min for staining, respectively. Fluorescence images were taken with a fluorescence microscope (DMI6000B, Leica, Germany) and analyzed by using Image J software.

The proliferation of rBMSCs on the scaffolds was assessed using a Cell Counting Kit (CCK-8) assay (Beyotime Institute of Biotechnology, Jiangsu, China). Briefly, 450 μl of culture medium and 50 μl of CCK-8 solution were added to each well on day 3 and 7 and incubated at 37 °C. Four hours later, an aliquot of 100 μl of each group was transferred to another 96-well plate and absorbance at 450 nm was measured with a microplate reader (Bio-Rad 680; Bio-Rad, Hercules, CA, USA).

### 2.6 Alkaline phosphatase (ALP) activity of rBMSCs cells on scaffolds

To determine the ALP activity of osteoblasts on scaffolds, 1 × 10^5^ cells were seeded on each scaffold and cultured in a 24-well plate for 7 and 14 days, respectively. At the predetermined time point, culture medium was removed and washed with PBS three times, followed by washing once in cold 50 mM Tris-buffer, and then cells were lysed in 200 μl of 0.2% Triton X-100. Lysates were sonicated after being centrifuged at 14,000 rpm for 15 min at 4 °C. Fifty microliters of supernatant was mixed with 150 μl working solution according to the manufacturer’s protocol (Jiangcheng, Nanjing, China). The results were obtained by measuring the absorbance at 405 nm with a microplate reader (Bio-Rad680). The ALP activity was calculated from a standard curve after normalizing to the total protein content, which was determined by the total protein assay kit (Jiangcheng, Nanjing, China) at 570 nm with a microplate reader (Bio-Rad 680). The results were expressed in μM of p-nitrophenol produced per min per mg of protein.

### 2.7 Osteogenic-related gene expression of rBMSCs on scaffolds

The relative expressions of osteogenic genes collagen type I (COL-1), osteocalcin (OCN), bone morphgenetic protein-2 (BMP-2), osteopontin (OPN), and bone sialoprotein (BSP) were measured by quantitative real-time PCR. Briefly, cells seeded at a density of 2 × 10^4^ on each scaffold were cultured for 7 or 14 days and harvested using Trizol Reagent to obtain RNA. The acquired DNA was reverse-transcribed into complementary DNA (cDNA) using Revert-Aid First Strand cDNA Synthesis Kit (Thermo) and the qRT-PCR analysis was performed on an ABI Prism 7300 Thermal Cycler (Applied Biosystems, Melbourne, Australia) using SYBR Green detection reagent. The relative expression of the osteogenic genes was normalized according to the housekeeping gene GAPDH. All samples were tested in triplicate and independent experiments were performed. The mean cycle threshold (Ct) value of each target gene was normalized against the Ct value of GAPDH. The relative expression was calculated using the following formula: 2^(-normalized average Ct)^ × 100.

### 2.8 Statistical analysis

Three separate experiments were carried out to collect the data, and then the data were expressed as means ± standard deviation. The level of significance was determined by the one-way ANOVA and Student-Newman-Keuls *post hoc* tests, and *p* values < 0.05 were considered to be significant.

## 3. Results

### 3.1 Fabrication and characterization of β-Ca_2_SiO_4_ scaffolds

**Figure 1.**
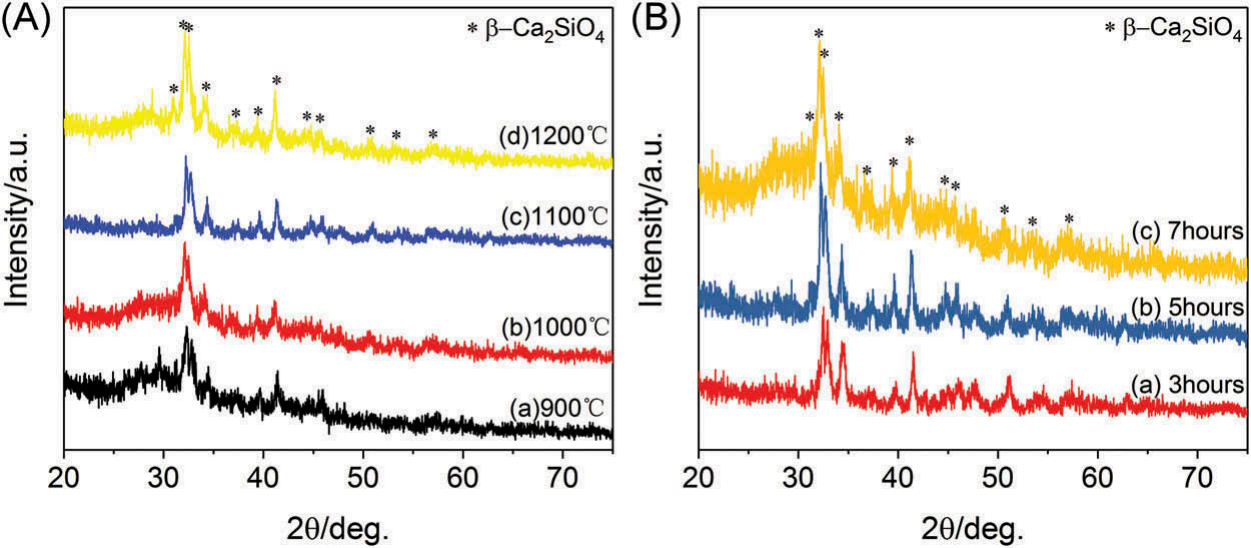
XRD patterns of (A) β-Ca_2_SiO_4_ scaffolds sintered at different temperature for 5h and (B) β-Ca_2_SiO_4_ scaffolds sintered at 1100 °C at different holding times.

The phase purity of the 3D-printed ceramic scaffolds sintered at different temperature was examined by XRD analysis. As shown in Figure 1, similar diffraction peaks are appeared for each type of scaffolds, and the characteristic peaks of these ceramic scaffolds can be assigned to β-Ca_2_SiO_4_ phase (JCPDS card 33-0302) with larnite structure. It can be clearly observed that β-Ca_2_SiO_4_ phase has formed at 900 °C and the crystallinity of the ceramics increased with increasing the sintering temperature. The crystallinity was calculated as 35.29%, 45.29%, 50.01%, and 64.85% for samples sintered at 900 °C, 1000 °C, 1100 °C, and 1200 °C, respectively. On the other hand, there was no obvious difference on the crystallinity when the scaffolds were sintered at 1100 °C for 3, 5, and 7 h, suggesting the reaction between CaCO_3_ and silicone was relatively fast due to homogeneous mixing.

**Figure 2.**
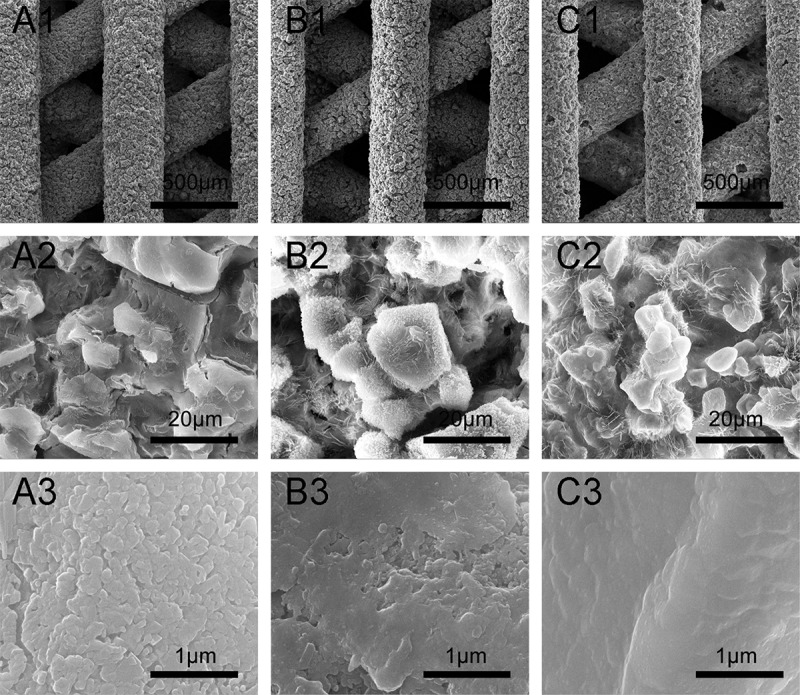
SEM images of β-Ca_2_SiO_4_ scaffolds sintered at 1000 °C (A1–A3), 1100 °C (B1–B3), and 1200 °C (C1–C3).

**Figure 3.**
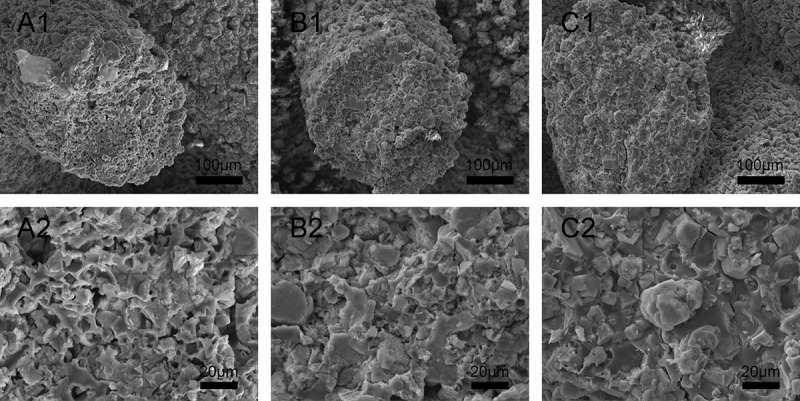
SEM images of fracture surface of β-Ca_2_SiO_4_ scaffolds sintered at 1000 °C (A1–A2), 1100 °C (B1–B2), and 1200 °C (C1–C2).

Figure 2 shows the surface morphologies of scaffolds sintered at 1000 °C, 1100 °C, and 1200 °C for 3 h. Each type of β-Ca_2_SiO_4_ scaffolds had same regular parallel pore structure, and the distance between two struts was about 400 μm, which exhibited about 9.3% shrinkage compared to green scaffolds. On the other hand, the surfaces of each type of scaffolds were rough, and the pores at microscale can be clearly observed on each strut. The densification degree of the scaffold strut increased with increasing sintering temperature, which is consistent with the crystallinity at different sintering temperature by XRD analysis. SEM images of fracture surface of β-Ca_2_SiO_4_ scaffolds sintered at 1000 °C, 1100 °C, and 1200 °C were shown in Figure 3. It could be observed that the crystallinity became higher and the porosity in the struts decreased, as well as the grain size grew with increasing the sintering temperature. The grain size of the scaffolds sintering at 1200 °C was estimated at 10 μm.

**Figure 4.**
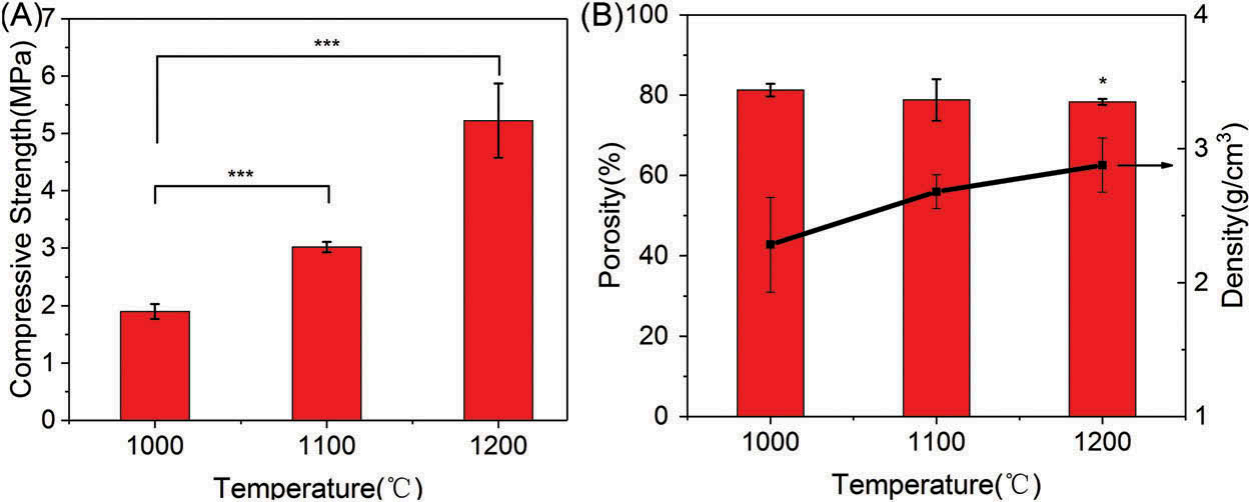
(A) Compressive strength and (B) porosity and density of β-Ca_2_SiO_4_ scaffolds sintered at different temperatures. (*n* = 3; * and *** indicate significant differences, *p* < 0.05 and *p* < 0.005).

The compressive strength, porosity, and density of 3D-printed β-Ca_2_SiO_4_ scaffolds are shown in Figure 4. The compressive strength of β-Ca_2_SiO_4_ scaffolds increased with sintering temperature and was 1.9 ± 0.1, 3.5 ± 0.8, and 5.2 ± 0.7 MPa for samples sintered at 1000 °C, 1100 °C, and 1200 °C, respectively. The porosity of β-Ca_2_SiO_4_ scaffolds is 81.3 ± 1.6%, 79 ± 5%, and 78.3 ± 0.7%, and the corresponding densities are 2.6 ± 0.4, 2.7 ± 0.3, and 2.9 ± 0.2 g/cm^3^.

### 3.2 Degradation and apatite mineralization ability of β-Ca_2_SiO_4_ scaffolds

**Figure 5.**
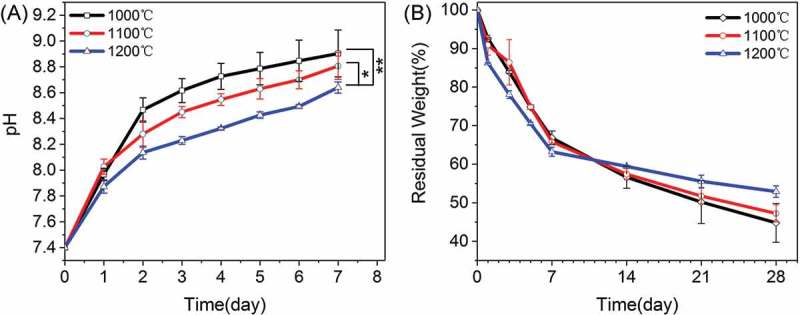
(A) pH values and (B) *in vitro* degradation of β-Ca_2_SiO_4_ scaffolds sintered at different temperatures in SBF (*n* = 3; * and ** indicate significant differences, *p* < 0.05 and *p* < 0.01).

Figure 5 shows the weight loss of each type of β-Ca_2_SiO_4_ scaffolds along with the pH changes in the microenvironment. It can be observed that the pH values of SBF increased with soaking time and decreased with the sintering temperature. After soaking for 7 days, the pH values for β-Ca_2_SiO_4_ scaffolds sintered at 1000 °C, 1100 °C, and 1200 °C were 8.9 ± 0.2, 8.8 ± 0.1, and 8.64 ± 0.04, respectively. Correspondingly, the weight loss was obviously occurred for each type of β-Ca_2_SiO_4_ scaffolds due to the degradability of β-Ca_2_SiO_4_ scaffolds, and the weight loss of β-Ca_2_SiO_4_ scaffolds increased with the decrease of sintering temperature. After soaking in SBF for 4 weeks, the residual weight of β-Ca_2_SiO_4_ scaffolds sintered at 1000 °C, 1100 °C, and 1200 °C were 45 ± 5, 47 ± 2, and 52.9 ± 1.5, respectively.

**Figure 6.**
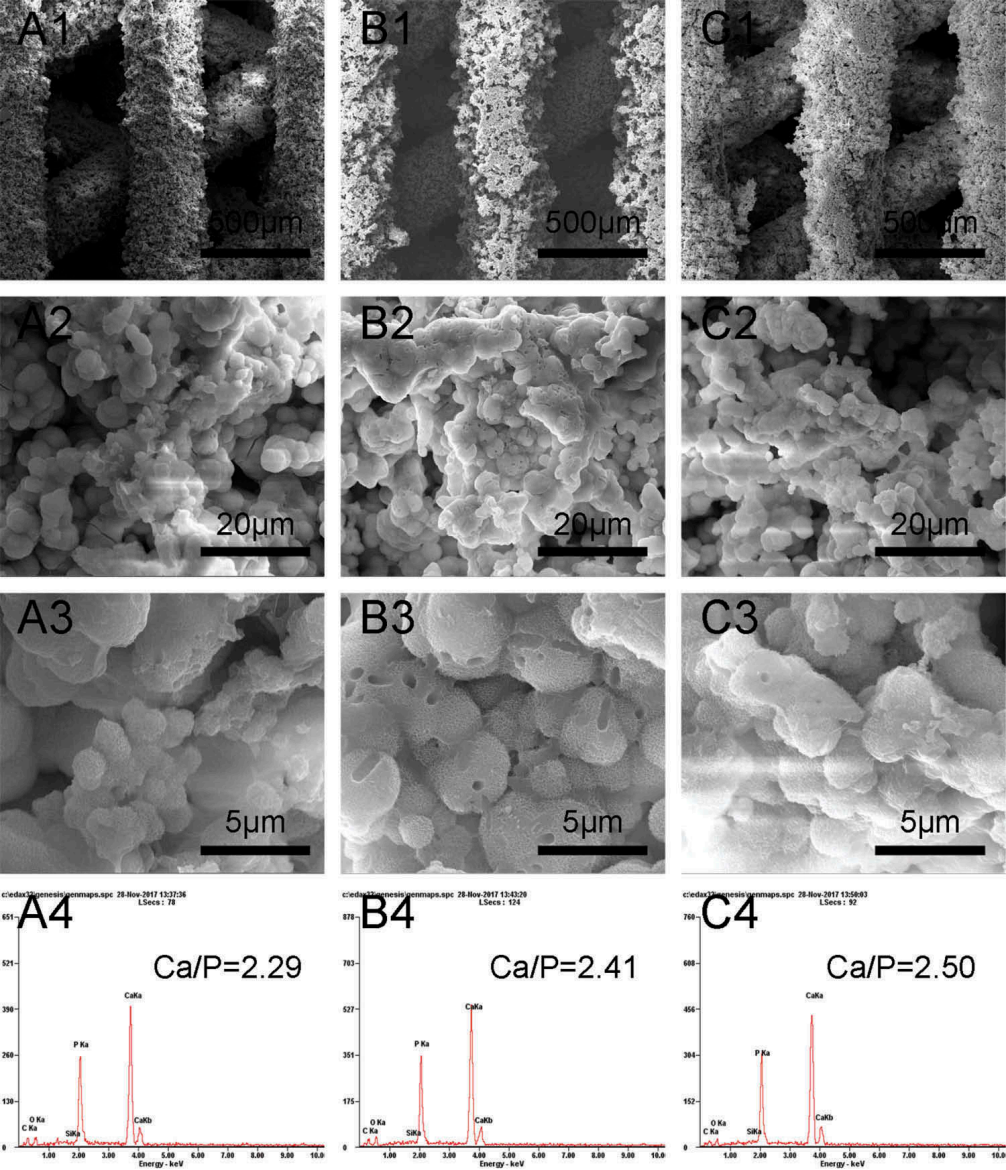
SEM images of β-Ca_2_SiO_4_ scaffolds sintered at (A1–A3) 1000 °C, (B1–B3) 1100 °C, and (C1–C3) 1200 °C after immersed in SBF for 7 days; EDS analysis of β-Ca_2_SiO_4_ scaffolds sintered at (A4) 1000 °C, (B4) 1100 °C, and (C4) 1200 °C after immersed in SBF for 7 days.

Apatite formation on β-Ca_2_SiO_4_ scaffolds after soaking in SBF was observed by SEM. As shown in Figure 6, all β-Ca_2_SiO_4_ scaffolds kept the interconnected macroporous structure after 7 days soaking in SBF, but the scaffold surface became much rougher and the spherical particles formed on the surfaces. EDS analysis indicated the presence of calcium, silicon, oxygen, and phosphorus. The Ca/P ratio of the surface of β-Ca_2_SiO_4_ scaffolds sintered at 1000 °C, 1100 °C, and 1200 °C were 2.29, 2.41, and 2.50, indicating the formation of like-apatite mineralization on each type of β-Ca_2_SiO_4_ scaffolds.

### 3.3 Cell responses to β-Ca_2_SiO_4_ scaffolds

**Figure 7.**
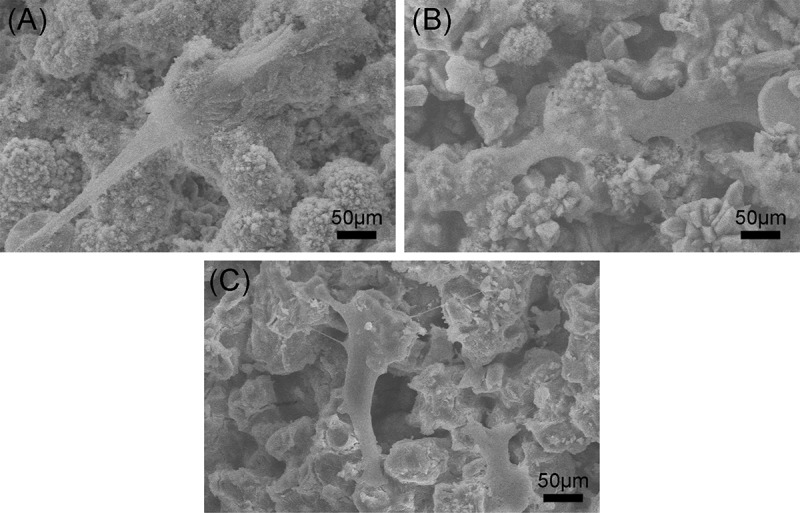
SEM images of the attachment of rBMSCs on β-Ca_2_SiO_4_ scaffolds sintered at (A) 1000 °C, (B) 1100 °C, and (C) 1200 °C after cell culture for 3 days.

**Figure 8.**
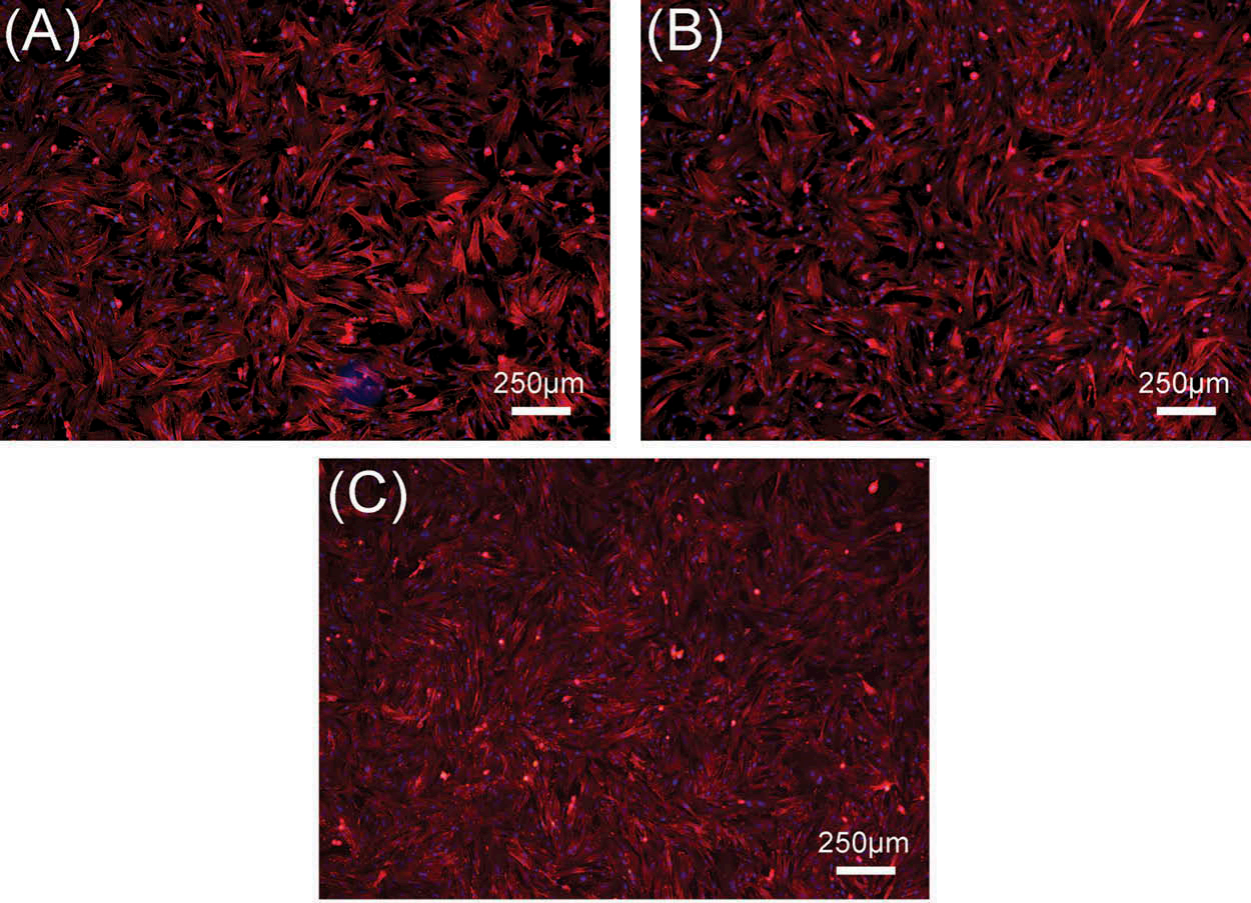
The fluorescence images of rBMSCs seeded on β-Ca_2_SiO_4_ scaffolds sintered at (A) 1000 °C, (B) 1100 °C, and (C) 1200 °C for 3 days. The cell actin and nuclei were labeled with phalloidin and DAPI.

In this study, rBMSCs were used to investigate cell responses to β-Ca_2_SiO_4_ scaffolds. Figure 7 shows the attachment and morphology of rBMSCs on β-Ca_2_SiO_4_ scaffolds sintered at 1000 °C, 1100 °C, and 1200 °C. After cultured for 3 days, rBMSCs were observed to be attached on the surface of the struts. rBMSCs exhibited a well-spread morphology on each type of scaffolds, and the density of rBMSCs on the scaffolds had no obvious difference. On the other hand, a cell fluorescent staining analysis was performed to investigate cell response to β-Ca_2_SiO_4_ scaffolds. Figure 8 shows fluorescence images of cells on β-Ca_2_SiO_4_ scaffolds struts after culture for 3 days. It can be observed that cells distributed homogeneously on all of scaffold struts with good cell spreading, suggesting the support of cell attachment. The amount of cells on the β-Ca_2_SiO_4_ scaffolds showed a little increase for the scaffolds sintered with higher temperature, which is inconsistent with that from the cell proliferation assay.

**Figure 9.**
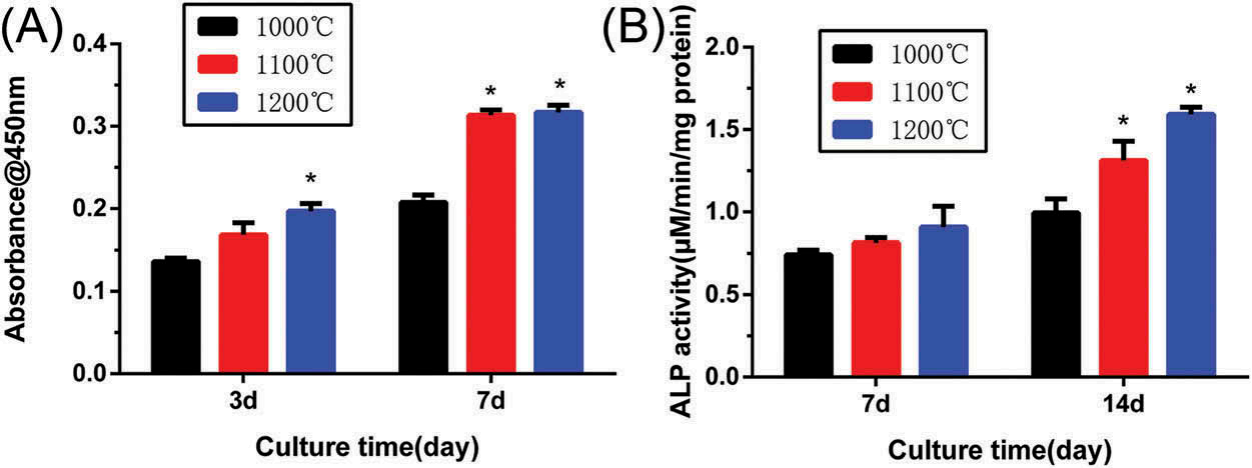
(A) Quantitative analysis of the proliferation of rBMSCs cultured on β-Ca_2_SiO_4_ scaffolds for 3 and 7 days (*n* = 3); (B) ALP activity of rBMSCs cultured for 7 and 14 days on β-Ca_2_SiO_4_ scaffolds for 7 and 14 days (*n* = 3). (* indicates significant differences when compared to β-Ca_2_SiO_4_ scaffolds sintered at 1000 °C).

Figure 9 shows the proliferation and ALP activity of rBMSCs on β-Ca_2_SiO_4_ scaffolds. The CCK-8 assay (Figure 9A) for 3 and 7 days revealed that cell proliferation increased over time, which indicates the scaffolds supported cell adhesion and viability. The proliferation of rBMSCs was significantly higher on the β-Ca_2_SiO_4_ scaffolds sintered at 1100 °C and 1200 °C than at 1000 °C (*p* < 0.05). ALP activity of rBMSCs cultured on β-Ca_2_SiO_4_ scaffolds for 7 and 14 days are shown in Figure 9B. Similar to the results of cell proliferation on the scaffolds, ALP activities of β-Ca_2_SiO_4_ scaffolds enhanced over time (*p* < 0.05) and the scaffolds sintered at 1100 °C and 1200 °C were significantly higher than that on the scaffolds sintered at 1000 °C at day 14.

**Figure 10.**
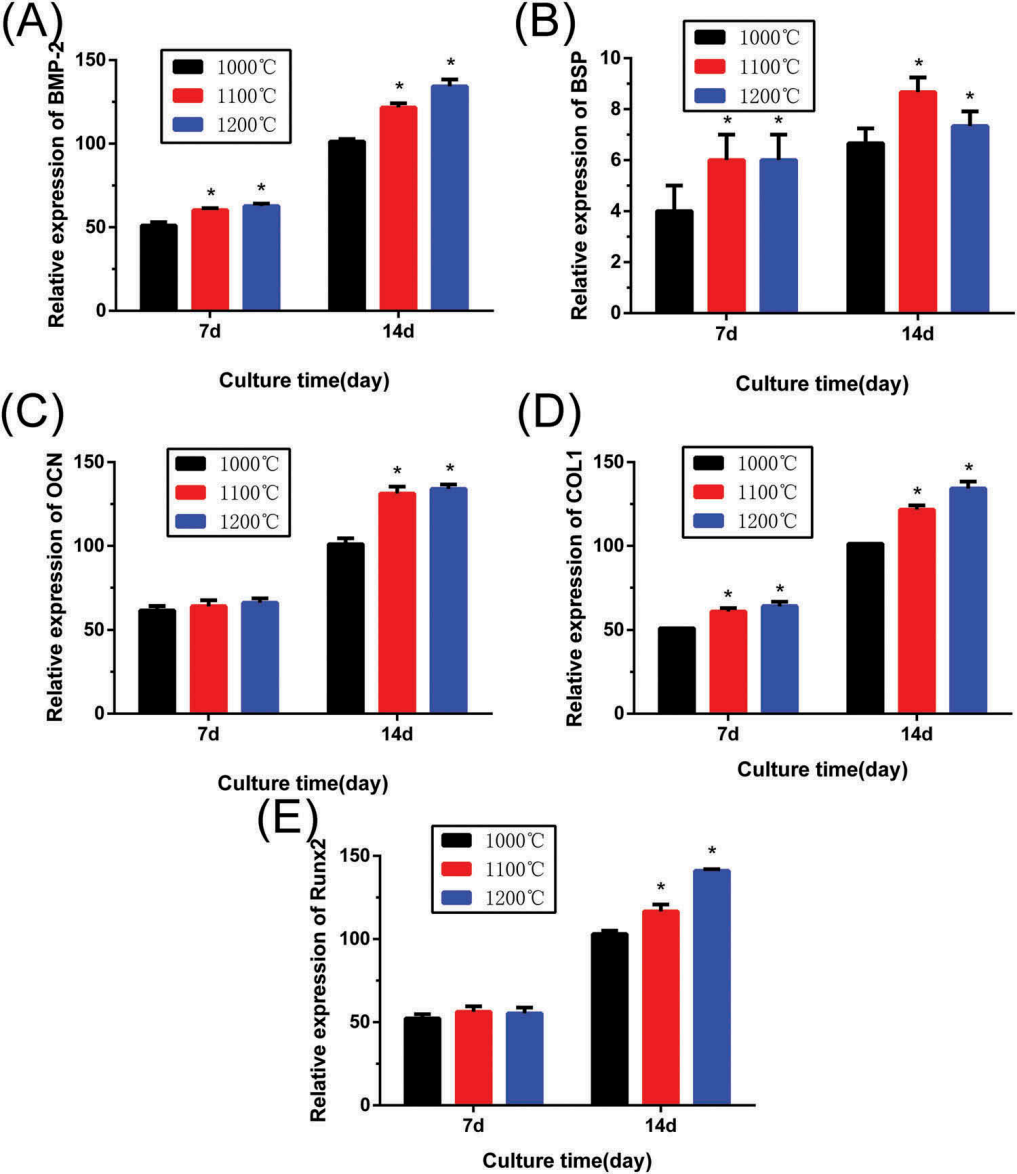
Osteogenic expression of (A) BMP-2, (B) BSP, (C) OCN, (D) OCL1, and (E) RUNX2 for rBMSCs cultured on β-Ca_2_SiO_4_ scaffolds by qRT-PCR analysis after 7 and 14 days (*n* = 3; * indicates significant differences when compared to β-Ca_2_SiO_4_ scaffolds sintered at 1000 °C, *p* < 0.05).

Cell differentiation of rBMSCs on β-Ca_2_SiO_4_ scaffolds was further assessed by osteogenic expression determined by the expressions of osteogenic markers BMP-2, BSP, OCN, COL-1, and RUNX2 at day 7 and 14 (Figure 10). Results of gene expression analysis showed that expression of all of osteogenic-related genes were upregulated on the scaffolds with increasing culture time. In addition, the increased sintering temperature for the scaffolds could promote osteogenic differentiation of rBMSCs on β-Ca_2_SiO_4_ scaffolds. Importantly, the scaffolds sintered at 1100 °C and 1200 °C exhibited significantly higher expression levels compared to the scaffolds sintered at 1000 °C (*p* < 0.05).

## 4. Discussion

In this study, we have successfully fabricated porous β-Ca_2_SiO_4_ scaffolds derived from a preceramic resin and CaCO_3_ active filler by 3D printing. A printable paste was prepared by dissolving the silicone resin (MK) as a binder and a reactant into isopropanol, and followed the addition of CaCO_3_ powders into the MK solution by stirring. Apparently, the preceramic silicone resin and CaCO_3_ active filler were mixed in a stoichiometric ratio 2:1 to form Ca_2_SiO_4_ according to the equation: 2CaO + SiO_2_ = Ca_2_SiO_4_. The 3D-printed scaffolds had regular and interconnected macropores after sintering at 1000−1200 °C, which maintain the 3D-printed structure of green scaffolds, suggesting the sintering process did not influence the macroporous structure. Generally, macroporous structure with a pore size larger than 150 μm facilitates cell attachment, migration, flow transport of nutrients, and bone ingrowth into scaffolds [42,43]. Therefore, the 3D-printed scaffolds had a desirable macroporous structure for application in bone tissue engineering.

According to the XRD analysis (Figure 1), β-Ca_2_SiO_4_ scaffolds can form above 900 °C due to the reaction between CaO and SiO_2_, which derived from CaCO_3_ and MK during sintering process. With the increase of sintering temperature from 900 °C to 1200 °C, the crystallinity of β-Ca_2_SiO_4_ was enhanced. The process of ceramic sintering is dependent on substance diffusion at a high temperature. With the increase of sintering temperature, the diffusion capacity of the reactants improved, leading to a high crystallinity. On the contrast, the poorer crystallinity of scaffolds sintered at lower temperature could be attributed to the inadequate reaction. However, the crystallinity of β-Ca_2_SiO_4_ scaffolds is not high due to the granularity of CaCO_3_ powders. As the granules size decreases, specific surface energy and thus crystallinity increase [38,41]. Actually, the crystallinity would be determined by sintering temperature and time except for particle size [44]. In this study, there was no significant difference in the crystallinity when the scaffolds were sintered at 1100 °C for 3, 5, and 7 h, suggesting the ceramization was relatively rapid, and sintering time exhibited less impact on ceramization than sintering temperature. On the other hand, β-Ca_2_SiO_4_ scaffolds had rough surface and pores on the struts, which might be the gas release that induced from the reaction during sintering process [2,3]. In fact, the rough surface is benefit for cell adhesion, differentiation and thereby formation of cell matrix [45–47]. Therefore, the 3D-printed preceramic polymer-derived scaffolds had a desirable phase of β-Ca_2_SiO_4_ and rough surface, which might be promising for bone tissue engineering.

Mechanical strength of ceramic scaffolds in bone tissue engineering also plays crucial roles [48,49]. As shown in Figure 4, the mechanical strengths of β-Ca_2_SiO_4_ scaffolds were remarkably enhanced by sintering temperature (*p* < 0.05) and an average compressive strength of 5.2 MPa actually was measured on the β-Ca_2_SiO_4_ scaffolds sintered at 1200 °C with a porosity of 78.3 ± 0.7%. With the increasing of sintering temperature, the scaffolds become more densification in accordance with the morphology in Figures 2 and 3. The increased crystallinity of the ceramics could be attributed to the better diffusion ability of reactants at higher sintering temperatures. Generally, a ceramic consists of crystal phase, amorphous phase, and pores, and the higher crystallinity at higher sintering temperature induces more density and less pores, which might contribute the enhanced compressive strength for β-Ca_2_SiO_4_ scaffolds sintered at 1200 °C compared to those scaffolds sintered at 1000 °C and 1100 °C. The results indicated that the β-Ca_2_SiO_4_ scaffolds meet the requirement of the compressive strength of cancellous bone (2–12 MPa) [48,50].

It has been accepted that pH microenvironment and degradation ability of the scaffolds exert direct influences to cell viability and proliferation. After soaking in SBF, Ca^2+^ ions were readily to release from the scaffolds and an increase of pH value occurred due to the production of OH^−^ induced from the hydrolysis of Ca^2+^ accompanied with the degradation of scaffolds [51]. However, β-Ca_2_SiO_4_ scaffolds sintered at 1200 °C were favorable to achieve a more balanced pH value in the microenvironment owing to the highest crystallinity. Lower crystallinity of the scaffolds implied greater dissolution, leading to more Ca^2+^ ions release as well as scaffolds degradation. With the increase of sintering temperature, the increased crystallinity slows down the degradation, and thereby results in a more stable pH variation [52]. The scaffolds had a relatively rapid degradation ability, which could be explained by the great degradation characters of β-Ca_2_SiO_4_ and the high porosity promoting the dissolution of the scaffolds [48].

The formation of apatite layer on the implanting materials surface is important for its binding to living bone tissue, and this bone binding ability can be evaluated *in vitro* in SBF [3,28]. After soaking in SBF for 7 days, the surfaces of the scaffolds were covered with spherical particles, and EDS analysis indicated the formation of like-apatite layer. The mechanism of apatite formation on the surface of β-Ca_2_SiO_4_ ceramics might be similar to that on bioactive glasses. Ca^2+^ ions in β-Ca_2_SiO_4_ interact with H_3_O^+^ ions in SBF, which could promote the formation of rich-Si layer on the ceramics. The rich-Si layer induces the formation of Ca–P nucleation and further apatite crystal formation [21,53]. In this study, the Ca/P ratios were 2.29, 2.41, and 2.50 for the scaffolds sintered at 1000 °C, 1100 °C, and 1200 °C, respectively, which are a little higher than that of 1.67 for apatite. The principle of EDS is dependent on the signal of characteristic X-ray. The characteristic X-ray just reflects the element species at a certain depth ranging from 500 nm to 5 μm. In this study, each type of β-Ca_2_SiO_4_ scaffolds degraded in SBF and formed spherical apatite particles on the surfaces. The Ca/P ratio of each β-Ca_2_SiO_4_ scaffold was a little higher than 1.67 for hydroxyapatite, which might be that the rich-Ca surface of the scaffold was also detected by EDS. On the other hand, there was no phosphorus element in the scaffolds, but phosphorus element was detected by EDS after soaking in SBF, which indicated the formation of apatite on the surface of the scaffolds.

Previous studies confirmed the good cytocompatibility for β-Ca_2_SiO_4_ materials [18,21,22]. Our study demonstrated that β-Ca_2_SiO_4_ scaffolds derived from preceramic polymer and CaCO_3_ active filler were able for cell adhesion, proliferation, and differentiation. rBMSCs on porous β-Ca_2_SiO_4_ scaffolds formed filopodia and lamellipodia after 3 days culture (Figure 7), suggesting the scaffolds support cell attachment. Meanwhile, porous β-Ca_2_SiO_4_ scaffolds stimulate cell proliferation and differentiation. However, the scaffolds sintered at higher temperature showed enhanced levels of cell proliferation and osteogenic-related gene expression, indicating that the sintering temperature could influence the biological property of scaffolds.

In this study, higher sintering temperature induced higher crystallinity of the β-Ca_2_SiO_4_ scaffolds. Lower crystallinity of the scaffolds implied greater dissolution, leading to more Ca^2+^ ions release as well as scaffolds degradation. Further, a weaker alkalinity microenvironment could be attributed to the hydration reaction between Ca^2+^ and H_2_O, which induced the nucleation of the hydroxyapatite crystals and the formation of apatite. On the other hand, the pH value of culture environment affects the cell’s behaviors and a much higher or lower pH value is harmful. Previous studies showed that a more stable pH value can stimulate metabolic activity, proliferation, and differentiation of osteoblasts [54,55]. In this study, the β-Ca_2_SiO_4_ scaffolds sintered at 1200 °C induced a more stable microenvironment, which might contribute the enhanced proliferation and differentiation of rBMSCs. Therefore, cell responses to the β-Ca_2_SiO_4_ scaffolds could be regulated by changing sintering temperature.

## 5. Conclusions

In summary, 3D porous β-Ca_2_SiO_4_ scaffolds have been successfully derived from a preceramic resin and CaCO_3_ active filler by 3D printing technique. The fabricated β-Ca_2_SiO_4_ scaffolds had uniform interconnected macropores (ca. 400 μm) and high porosity (>78%), and compressive strength was about 5.2 MPa for rectangular structures in scaffolds sintered at 1200 °C. Moreover, β-Ca_2_SiO_4_ scaffolds exhibited good apatite-forming ability in SBF. Importantly, the results indicated that the increase of sintering temperature significantly enhanced the compressive strength and the scaffolds sintered at higher sintering temperature stimulated the adhesion, proliferation, ALP activity, and osteogenic-related gene expression of rBMSCs. Therefore, the 3D-printed β-Ca_2_SiO_4_ scaffolds derived from preceramic resin and CaCO_3_ active fillers would be promising for bone tissue engineering.
